# Cross-Cultural Perspectives on Metabolic Syndrome Management and Attitudes Toward Using Digital Health Tools in Two Distinct Populations: Protocol for a Qualitative Descriptive Study

**DOI:** 10.2196/91903

**Published:** 2026-06-24

**Authors:** Asma Ali, Ifeanyichukwu Ogbuiyi-Chima, Mohammad Faisal, Noof Alwatban, Maryam Alhabas, Ashraf A El-Metwally, Ashish Joshi

**Affiliations:** 1 School of Public Health University of Memphis Memphis, TN United States; 2 Department of Public Health College of Public Health and Health Informatics King Saud Bin Abdulaziz University for Health Sciences Riyadh Saudi Arabia

**Keywords:** metabolic syndrome, self-management, digital health, qualitative research, cross-cultural comparison, chronic disease

## Abstract

**Background:**

Metabolic syndrome (MetS) is defined by the presence of at least 3 out of 5 clinical risk factors, including abdominal obesity, elevated blood pressure, high fasting glucose, elevated triglycerides, and low high-density lipoprotein cholesterol. Individuals with MetS face significantly increased risks of cardiovascular disease, type 2 diabetes, and all-cause mortality.

**Objective:**

This study aims to explore the multilevel factors influencing individuals’ engagement in self-management of MetS. Specifically, it seeks to identify key barriers and facilitators to effective self-management and to assess participants’ attitudes toward the use of digital health tools in supporting disease management.

**Methods:**

This qualitative descriptive study will be conducted at 2 international sites: the University of Memphis (United States) and King Saud bin Abdulaziz University for Health Sciences (Saudi Arabia), in collaboration with local primary care clinics. Participants will be recruited using purposive sampling with a maximum variation strategy, aiming for 20 to 30 individuals per site who meet eligibility criteria. Data will be collected through semi-structured, 60-minute one-on-one interviews. An abductive thematic analysis approach (integrating both inductive and deductive reasoning) will be used to analyze the data with NVivo 15 software.

**Results:**

This study will begin participant enrollment in August 2026. Thematic analysis will be used to examine barriers and facilitators to MetS self-management and participants’ views on digital health tools across 2 international sites.

**Conclusions:**

Findings will offer culturally grounded insights into how social, dietary, and familial factors influence chronic disease management. This knowledge can inform the design of equitable, context-sensitive digital health interventions.

**International Registered Report Identifier (IRRID):**

PRR1-10.2196/91903

## Introduction

### Background

Metabolic syndrome (MetS) is the co-occurrence of at least 3 out of 5 risk factors, as established by the National Cholesterol Education Program Adult Treatment Panel III (NCEP ATP III) [1,2]. The 5 risk factors are abdominal obesity (waist circumference >102 cm [>40 inches] in men or >88 cm [>35 inches] in women), elevated blood pressure (≥130/85 mm Hg or use of antihypertensive medication), high fasting glucose (≥100 mg/dL or previously diagnosed diabetes), elevated triglycerides (≥150 mg/dL), and low high-density lipoprotein cholesterol (<40 mg/dL in men or <50 mg/dL in women) [1]. MetS significantly elevates the risk of cardiovascular diseases (2-fold in men), type 2 diabetes (>4-fold), and all-cause mortality [3,4].

The overall prevalence of MetS in the United States is 39.8% among adults (40.6% in men and 39.1% in women) [5]. The weighted prevalence varies significantly by age (22% in young adults and 56% in people aged 60 years or older), ethnicity (highest in Hispanic populations), and socioeconomic status (higher among those with lower educational attainment and income) [5].

The global prevalence of MetS shows significant regional variations, reflecting differences in urbanization, lifestyle, and socioeconomic development [6]. The Middle East faces a substantial burden of MetS, with a reported prevalence ranging from approximately 15% to more than 60%, depending on the country, diagnostic criteria, and population subgroups [7]. For example, MetS prevalence estimates are 33.6% in Palestine and 44.1% in Jordan [8,9]. Using the NCEP ATP III criteria, the prevalence of MetS in Saudi Arabia is 39.9% (45% in men and 35.4% in women) [10]. In the Asia-Pacific region, the prevalence of MetS ranges from 12% to 37% based on national surveys, with higher rates typically observed in urban areas and among women [11]. In South Asia, a systematic review noted prevalence rates as high as 26.1% [12]. In Africa, the prevalence of MetS varies substantially depending on diagnostic criteria, population setting, and comorbid conditions, with reported ranges from 12% in Ethiopia to more than 50% among some urban and hypertensive populations in Nigeria [13]. In Latin America, the urban prevalence of MetS in large cities ranges from 14% in Quito to 27% in Mexico City [6]. On the basis of data from adults in 10 European countries, the prevalence of MetS is 24.3% [14]. These regional disparities underscore the need for culturally and contextually tailored public health interventions and the development of standardized diagnostic criteria to monitor and address the burden of MetS more effectively.

Social determinants of health significantly shape MetS outcomes. The World Health Organization defines social determinants as the conditions in which people are born, grow, work, live, and age [15]. Lifestyle and environmental factors, such as diet, physical activity, sedentary habits, and socioeconomic conditions, are widely recognized as predisposing risks to MetS [16-19]. Socioeconomic disparities intensify these challenges and make engagement in and sustainability of lifestyle changes more difficult [5,19,20]. For instance, people in the United States with lower income face cost barriers that may contribute to transportation difficulties and limited access to healthy foods and preventive care [5,19,20]. A recent study by Ma et al [18] found that adults with MetS who experienced the highest levels of cumulative social disadvantage faced more than twice the risk of cardiocerebrovascular mortality compared with those with the least disadvantage. Social disadvantage was assessed across 5 domains of social determinants of health, including economic stability, education, health care access, neighborhood environment, and social context [18]. In Saudi Arabia, rapid urbanization and modernization have contributed to decreased physical activity and a shift away from traditional dietary patterns toward the consumption of energy-dense and nutrient-poor foods [21,22]. These lifestyle changes represent key risk factors associated with the rising prevalence of chronic diseases [21-23]. Cultural traditions in Saudi Arabia emphasizing hospitality and shared meals can encourage overeating, while family expectations, social norms, and health literacy challenges shape dietary behaviors and readiness to adopt healthier lifestyles [24].

Effective and sustainable management strategies, including lifestyle changes (dietary modification, increased physical activity, and weight control) are essential for managing and preventing MetS [20,25]. Digital health tools (eg, mobile health apps, wearable trackers, SMS text reminders, and telehealth coaching) are increasingly being integrated into chronic disease management models [26,27]. These technologies offer scalable and accessible solutions to support sustained behavior change [26,27]. Their growing adoption reflects a shift toward more personalized and proactive approaches to health care delivery [28,29]. Digital health interventions have been associated with improved glycemic control, weight, waist circumference, and blood pressure among adults with chronic conditions such as type 2 diabetes [25]. However, most research has focused on high-income settings, with limited inclusion of lower-income and ethnically diverse populations [26,27,30,31].

Qualitative research highlights critical barriers to using digital health tools, including digital literacy, limited access to smartphones or reliable internet access, lack of culturally tailored content, privacy concerns, and varying levels of trust in technology across different countries [27,29,32]. Martinez Leal et al [33] reported that US Hispanic adults preferred culturally relevant, bilingual content that aligned with traditional dietary practices and family-centered values. This highlights the importance of tailoring interventions to cultural contexts to ensure true patient-centered care [33]. Age has also been found to be associated with acceptability of digital health tools; for instance, older adults with MetS may encounter barriers to digital engagement due to low health literacy and preference for in-person guidance [32]. These factors should be considered when designing evidence-based digital health interventions to ensure that they are culturally and contextually responsive to support equitable access, engagement, and ultimately improved health outcomes.

Despite the promise of digital health for supporting MetS management, significant barriers to self-management and adoption of these tools remain underexplored, particularly in culturally diverse international settings, such as within the United States and the Middle East [24,34]. Self-management practices can vary substantially across populations due to differences in health beliefs, social norms, economic status, and health care infrastructures [24,35]. This underscores the need for qualitative research to investigate barriers and facilitators to MetS self-management and to gain a better understanding of perceptions and use of digital health tools in diverse cultural contexts. Such research will be essential for guiding the design of equitable, culturally responsive, and practically effective digital interventions. Therefore, this qualitative study aims to explore cross-cultural perspectives on MetS self-management by examining 2 socioculturally distinct populations: adults residing in the United States and adults in the Middle East (with an emphasis on Saudi Arabia).

### Conceptual Models

To fully understand the complex and interdependent factors influencing the self-management of MetS, this study adopts the ecological model as a framework of inquiry. The ecological model considers self-management behavior as a product of dynamic interactions across multiple levels of influence [36]. These include individual-level factors (eg, motivation and digital literacy), interpersonal factors (eg, family and peer support), and broader structural forces (eg, health care infrastructure and sociocultural norms) [31-33,36]. While the ecological model has been used in various forms, the framework applied here focuses on how contextual environments shape personal health practices and technology engagement [31,37]. For instance, barriers such as a lack of digital skills or time constraints exist at the individual level, while facilitators such as family encouragement and culturally tailored interventions emerge at the interpersonal and community levels [33,38]. The ecological lens supports a holistic inquiry into how personal, social, and environmental determinants influence behavior, making it particularly valuable for understanding the uptake and impact of digital health tools across culturally diverse populations.

The capability, opportunity, motivation–behavior (COM-B) model is used to guide the understanding of the whole system behind behavioral change needs [39]. This model will help to improve intervention design by understanding the nature of people’s attitudes toward the use of digital tools for the management of MetS. This understanding will help characterize interventions and their components [39]. Therefore, this will be a starting point for understanding the different types of interventions that are likely to be effective as a basis for intervention design [39]. The COM-B system suggests that capability (C), opportunity (O), and motivation (M) interact to generate a behavior (B) [39]. Capability includes an individual’s psychological and physical capacity to engage in the behavior [39]. Motivation includes habitual processes, emotional responding, and analytic decision-making processes that support the engagement in a behavior [39]. Opportunity focuses on outside factors that make the behavior possible to engage in [39].

### Aims and Research Questions

This study aims to understand the multilevel factors that shape individuals’ engagement in MetS self-management. In particular, the study focuses on finding barriers and facilitators to effective self-management and evaluating attitudes toward using digital health tools to support disease self-management. To address these objectives, the following research questions guide this study.

- Question 1: What are the key barriers and facilitators to MetS self-management among individuals in the United States and the Middle East? This question seeks to identify individual, interpersonal, organizational, and community factors influencing self-care practices.
- Question 2: What individual, interpersonal, and community influences shape the adoption and use of digital tools for MetS management? This question investigates how family dynamics, religious practices, traditional beliefs, and socioeconomic realities mediate the effectiveness of digital health interventions [19,40].
- Question 3: How do attitudes toward digital health tools vary between the 2 populations, and what factors shape their acceptance or rejection? This question will explore capabilities, opportunities, and motivations in relation to perceptions of technology usefulness, ease of use, trustworthiness, and cultural relevance, especially in light of challenges such as low digital literacy, infrastructure gaps, and trust issues [32,39,41].

## Methods

### Study Approach

This study uses a qualitative descriptive methodology to explore participants’ experiences in managing MetS, including the factors that facilitate or hinder their management, as well as their engagement with digital health technologies [42]. The study will be conducted across 2 international collaborating sites: The University of Memphis in the United States, in partnership with a local primary care clinic, and King Saud bin Abdulaziz University for Health Sciences (KSAU-HS) in Saudi Arabia. Qualitative descriptive methodology was selected to allow for an in-depth, practice-oriented understanding of participants’ lived experiences without imposing restrictive theoretical interpretation, which is appropriate for the cross-cultural exploration of chronic disease self-management and digital health engagement [42,43].

### Participants

Participants will be eligible for inclusion in the study if they meet one of the following 2 criteria for having MetS:

- Criterion 1: a documented diagnosis of MetS by a physician, identified through the *International Classification of Diseases, Tenth Revision* code E88.81.
- Criterion 2: at the time of recruitment, participants must exhibit at least three of the following five risk factor categories: (1) abdominal obesity defined as a waist circumference >102 cm (>40 inches) in men or >88 cm (>35 inches) in women for people in the United States and a waist circumference >94 cm (>37 inches) in men or >80 cm (>31.5 inches) for people in Saudi Arabia; (2) elevated blood pressure (≥130/85 mm Hg or use of antihypertensive medication); (3) high fasting glucose (≥100 mg/dL or previously diagnosed diabetes); (4) elevated triglycerides (≥150 mg/dL); (5) low high-density lipoprotein cholesterol (<40 mg/dL in men and <50 mg/dL in women). Waist circumference thresholds will be studied site specifically to account for cross-cultural differences in the population; participants in the United States will follow the NCEP ATP III threshold, and participants recruited in Saudi Arabia will follow the International Diabetes Federation recommendations for Middle Eastern populations [1,44].

### Participant Recruitment

A designated clinic staff member from each site (United States and Saudi Arabia) will identify eligible participants who meet the predefined inclusion criteria, either through a review of the electronic health records or via referral from the health care professional during routine clinic visits. Individuals who meet the eligibility criteria will be approached by clinic staff, who will provide information about the study. If the individual expresses interest in taking part, the staff will obtain written permission to share their contact information with the research team. Once permission is granted, the clinic staff will forward the contact information to the designated research team member at each site. Then, the research team will contact the participant to schedule the interview and obtain formal informed consent, emphasizing that participation is completely voluntary. To further minimize potential power dynamics between patients and clinic staff, clinic personnel will not be involved in obtaining informed consents or conducting interviews.

### Sampling

A purposive sampling technique using a maximum variation strategy (eg, age, gender, and duration of illness) will be used to recruit participants [45]. Purposive sampling involves selecting participants who have experience with the phenomenon of interest, which is MetS self-management in this research study [45]. The maximum variation strategy is used as eligible participants will be recruited from partnering clinics in 2 distinct populations [45]. On the basis of information power, the total number of participants needed for this study can range between 20 and 30 people from each country, with a total of 40 to 60 participants in the study [46]. Information power is based on five main components with the following characteristics needed for a lower number of participants: (1) narrow study aim, (2) sample specificity (participants with relevant experience), (3) use of established theory, (4) strong in-depth interviews, and (5) in-depth analysis strategy [46].

Information power will be assessed iteratively, with attention to both within-site and cross-site variations to ensure the attainment of sufficient depth of information to identify shared and context-specific themes across the United States and Saudi Arabia [44]. Recruitment will continue within each site until sufficient information is gained to answer our research questions, defined as the point at which no new substantive themes emerge within and across sites [46].

### Data Collection and Interview Procedure

To gather qualitative data, semi-structured, one-on-one interviews lasting approximately 60 minutes will be conducted using the developed interview protocol (Multimedia Appendix 1). The interview guide includes a brief explanation of digital health tools (eg, mobile apps, wearable devices, telehealth platforms, SMS text message–based support, and patient portals) and domains, such as perceived usefulness, usability, access or feasibility, and cultural fit, to ensure conceptual clarity across participants. Trained interviewers will carry out the interviews in a private meeting room located either at the designated university site (The University of Memphis or KSAU-HS) or at the partnering clinic site. Interviews will be conducted in a confidential setting, with the presence of an interpreter only when necessary to facilitate communication. When preferred by participants or required for logistical reasons, interviews may also be conducted virtually via Microsoft Teams or by phone using the institution’s landline.

All interviews will be audio-recorded using digital recording equipment, such as a Yeti microphone connected to a university-owned, password-protected laptop. The recordings will be securely stored in a university-supported, encrypted Microsoft OneDrive folder. Access to the recordings will be restricted to authorized members of the research team and individuals directly involved in transcription and translation. Verbatim transcription will be carried out by either a trusted professional transcription service or a trained student research assistant.

### Interpretation and Translation

Interviews conducted in Saudi Arabia will be administered in Arabic, using a translated version of the interview protocol. To ensure linguistic and conceptual equivalence, both the protocol and the verbatim transcripts will undergo a rigorous translation and back-translation process, as recommended in cross-cultural research methodologies [47]. Interviews carried out in the United States will be conducted in English. For participants in the United States who prefer to communicate in Spanish, a professional interpreter will facilitate the sessions using a triadic communication model (interviewer →interpreter → interviewee, and vice versa), ensuring accurate and culturally sensitive exchange throughout the interview process. The interview protocol will be shared with the interpreter in advance to allow sufficient time for familiarization and to support consistency in interpretation.

### Theoretical Framework and Cross-Cultural Lens

This study is guided by the ecological model and the COM-B framework [36,39]. These frameworks support the examination of MetS self-management and digital health engagement at the individual, interpersonal, organizational, and cultural levels. The ecological model will guide the deductive analysis of the first 2 research questions, which focus on barriers and facilitators to MetS self-management and the individual, interpersonal, and community influences shaping the adoption and use of digital tools for MetS management.

The COM-B model will guide the analysis of the third research question, which examines attitudes toward digital health tools and the factors shaping their acceptance or rejection. This analysis will enable the research team to look at the existing capabilities related to using digital tools in these 2 different populations and identify opportunities for improvement based on people’s intrinsic and extrinsic motivations [39].

### Analysis Plan

This study will use an abductive thematic analysis approach, combining both inductive and deductive methods [48,49]. The deductive component will be informed by the ecological and COM-B models, which will serve as frameworks for organizing the overarching themes [36]. Simultaneously, the inductive approach will allow for the identification and integration of any emergent themes that may arise directly from the data [49].

Data analysis will be conducted by two trained researchers per site who will follow these steps: (1) familiarization with data, (2) generating initial codes, (3) searching for themes, (4) reviewing themes, (5) defining and naming themes upon consensus, and (6) producing the report [48]. NVivo software (version 15; Lumivero) will be used to analyze the data.

The 2 researchers at each site will independently code an initial subset of transcripts (approximately 20%) to assess coding consistency across analysts. Discrepancies will be discussed and resolved through consensus. A shared codebook will be developed and refined iteratively throughout the analysis process to promote consistency across sites. The data analysis teams from both sites will meet regularly to review the initial codebook, discuss similarities and differences in coding, and resolve discrepancies. Throughout this process, the researchers will remain mindful that certain themes may emerge in one country but not the other. The research team will maintain reflexive memos throughout data collection and analysis to document positionality, assumptions, and analytic decision-making, particularly in relation to cross-cultural interpretation. The study will be reported in accordance with the Consolidated Criteria for Reporting Qualitative Research (COREQ) [50].

### Ethical Considerations

Institutional review board (IRB) approval will first be obtained from the University of Memphis for all study sites and procedures. Following this initial approval, IRB clearance will be sought from KSAU-HS, as well as from its partnering clinic if required. Additional IRB approval from partnering clinics will be obtained if required by institutional policy.

Possible risks include discomfort when discussing personal health experiences. Given the cross-cultural nature of the study, interviewers will conduct discussions with sensitivity to cultural norms related to diet, family roles, and health beliefs. Participants may decline to answer any question or discontinue participation at any time without giving a reason and with no impact on their clinical care. All data will be deidentified prior to analysis and securely stored to protect confidentiality, including during cross-country data transfer. Access to identifiable data will be confined to authorized members of the research team, and no identifiable information will be shared between study sites. Recordings will be deleted once the transcripts have been validated by the research team and it has been determined that there is no need to retain them, which will occur within 1 year of the start of the study.

## Results

IRB approval is anticipated to be obtained from the University of Memphis by the end of July 2026, followed by local IRB approval from KSAU-HS and partnering clinics as required, with participant enrollment is scheduled to begin in August 2026 and will continue until sufficient information power is achieved and no new substantive themes emerge. Data collection will start immediately upon participant recruitment. Data familiarization will begin soon after receiving the written transcripts, and data will be analyzed starting in November 2026 using thematic analysis, supported by NVivo software (version 15). The findings from this study are expected to provide valuable insights into the unique barriers and facilitators influencing self-management of MetS across 2 international populations. Additionally, the study will explore participants’ perspectives on the use of digital health technologies in managing their condition, contributing to the development of culturally informed and contextually relevant interventions.

## Discussion

The significance of this inquiry lies in its potential to provide culturally nuanced insights that can inform tailored, patient-centered intervention designs. Evidence shows that cultural dietary habits, health beliefs, and familial structures deeply influence patients’ willingness and ability to adopt lifestyle changes and the use of digital tools for chronic disease management [32,33]. Understanding these dynamics across different contexts will contribute to the global health literature and support the development of equitable digital health interventions sensitive to local realities.

Building on this foundation, this study aims to identify multilevel factors that influence MetS self-management and engagement with digital health technologies in 2 distinct health care and sociocultural settings. These factors are anticipated to arise at the individual level (eg, health knowledge, motivation, and technological proficiency), the interpersonal level (eg, family support and prevailing social norms), and the health care system level (eg, access to care, health care provider communication, and the availability of digital tools) [22,25,31,33,36,39-41]. Comparative analysis between the United States and Saudi Arabia is expected to reveal both common challenges and context-specific influences shaped by cultural differences and health care delivery systems.

This study offers several strengths, such as its cross-cultural design, application of theory-informed qualitative methods, and recruitment of participants from real-world clinical environments. Nonetheless, certain limitations must be acknowledged. As a qualitative investigation, the findings will not be statistically generalizable, and despite the implementation of rigorous translation procedures, some linguistic or cultural nuances may not be fully captured. As a protocol, this manuscript describes planned methodologies rather than presenting empirical results. Furthermore, the use of site-specific waist circumference thresholds may limit strict comparability across study sites, however, it may enhance contextual and clinical relevance within each setting.

## Appendix

Multimedia Appendix 1 Interview protocol.

## Abbreviations

COM-B: capability, opportunity, motivation–behavior
COREQ: Consolidated Criteria for Reporting Qualitative Research
IRB: institutional review board
KSAU-HS: King Saud bin Abdulaziz University for Health Sciences
MetS: metabolic syndrome
NCEP ATP III: National Cholesterol Education Program Adult Treatment Panel III
