# Supplementary material for: Cross-Cultural Perspectives on Metabolic Syndrome Management and Attitudes Toward Using Digital Health Tools in Two Distinct Populations: Protocol for a Qualitative Descriptive Study
Source: JMIR Res Protoc. 2026 Jun 24;15:e91903. doi: 10.2196/91903 (PMC13347078; doi:10.2196/91903)
Supplement: Multimedia Appendix 1 [file resprot_v15i1e91903_app1.docx]

**Appendix A. Interview Protocol**

**Opening Script**

Thank you for meeting with me today! This interview aims to understand your personal experiences managing Metabolic Syndrome and your thoughts on using digital health tools. We are interested to learn from your experience and value what you share.

Consent form script: I will be recording this interview using [recording device]. After our interview, the research team will make a transcript of what you said, but we will remove your name and any names or locations you mention so nothing you say can be traced back to you. Also, we ask if you could refrain from mentioning specific names during the interview. Only the research team including myself will have access to the data, which will be stored securely on folders protected by “…”. If you'd like to skip a question or stop the interview at any point, that's okay; there is no judgment or penalty.]

Before we begin, do you have any questions? [Wait for questions. Answer them until the participant is ready.]
This interview will take about 60 minutes. Are you willing to start and be recorded now?
[Wait for confirmation.]
Great! I’ve started the recording. Can you confirm that you understand and give your consent to being recorded? [Wait for confirmation.]
Thank you! Let’s begin.

I will start with some general questions:

Have you heard the term metabolic syndrome?

1. Have you ever been told by a doctor that you have metabolic syndrome?
   1. *What other chronic conditions have your provider told you that you have? (e.g., high blood sugar, high blood pressure…)*
2. What do you know about metabolic syndrome?
   1. *What is it?*
   2. *What do you know about managing it?*
3. Do you use a smartphone, a tablet, or a computer? (Yes/No)
4. Have you ever used a mobile health application, health-related device or a digital platform? (Yes/No)
   1. *Tell me more about it? Please describe it.*
   2. *What did you use it for?*
   3. *Are you still using it? Why not?*

*Some questions about Familiarity with Technology might be important*

1. *Do you currently have a smart phone*

- *Basic phone*
- *Android Phone*
- *Apple*
- *Other, please specify*

*Do you have internet access on the phone*

- *Yes*
- *No*

*Do you use any health apps to currently manage your health condition*

- *Yes*
- *No*

*If yes, can you specify which apps and for what purpose*

*What are your primary sources of health information? (select all that applicable)*

- *Internet*
- *Social Media*
- *Family/Friends*
- *Physician*
- *Books/Magazine*
- *Other, please specify*

*Would you be interested in receiving health information over the phone*

- *In the form of app*
- *As a text message*
- *Video*
- *Other, please specify*

*How familiar are you with use of computers?*

- *Very familiar*
- *Somewhat familiar*
- *Not familiar at all*

**Interview Questions - Metabolic Syndrome Self-Management**

The next set of questions focus on understanding your experience in managing your metabolic syndrome and the factors that may make it easier or harder for you to take care of it.

1. Can you describe to me how do you take care of your metabolic syndrome?
   1. *What do you do to take care of it? (Individual level)*
   2. *How often do you do those [activities]? (Individual level)*
   3. *What helps you stick with them? (Individual, interpersonal, and organization levels)*
   4. *What gets in the way? (Individual, interpersonal, and organization levels)*
2. What are your goals from taking care of your metabolic syndrome? *(Individual, interpersonal levels)*
3. What makes it easier for you to take care of your metabolic syndrome? *(Individual, interpersonal, and organization levels)*
   1. *Tell me more about it.*
   2. *Can you give an example?*
   3. *What helps you the most? How?*
   4. *Who helps you the most (thinking of family members, thinking of healthcare providers and clinics)?*
      1. *How?*
      2. *How do you wish that they would support you in a way that is helpful for you?*
   5. *How do you feel that these factors are impacting your [goals mentioned in response to previous question] that you wanted to achieve?*
4. What makes it harder for you to take care of your metabolic syndrome? *(Individual, interpersonal, and organization levels)*
   1. *How about things in your daily to day life?*
      1. *How? Please explain more.*
   2. *How do you feel that these factors are impacting your [goals mentioned in response to previous question] that you wanted to achieve?*
   3. *What do you do to help you overcome those challenges?*
   4. *What do you wish were different?*
      1. *How do you wish that these challenges can be resolved?*
5. Have you received a treatment plan from your healthcare provider to manage your metabolic syndrome? Tell me more about it. *(Interpersonal, and organization levels)*
   1. *What makes it easier for you to follow your treatment plan? How?*
   2. *What makes it harder for you to follow your treatment plan? How?*
6. What do you think about the care you get from your clinic, doctor, or healthcare system? *(Interpersonal, and organization levels)*
   1. *How do they support you?*
   2. *Do they provide any services to help you overcome any of the things that makes it hard for you to take care of your metabolic syndrome [Probe the barriers mentioned before]?*
   3. *Are there things that you think they should provide to help you in taking care of your metabolic syndrome? Please explain more.*
   4. *What are the things that would make your experience better with your healthcare provider or clinic?*

**Interview Questions - Adoption and Utilization of Digital Tools**

The following questions focus on understanding your experience in using or knowing about any digital tools that could help in taking care of metabolic syndrome and the factors that may make it easier or harder for using it. A digital tool is something you can use on your phone, tablet, or computer to help you manage your health. It could be a mobile app, a website, or even a device that tracks your symptoms or medications. For example, if you have diabetes, a digital tool might help you keep track of your blood sugar levels, remind you to take your medicine, or show you tips for healthy eating. These tools are made to make life a little easier and help you stay on top of your condition, even between doctor visits.

1. Have you ever used a digital tool like a mobile health application, health-related device, digital tracker, or a digital platform to help you in taking care of your metabolic syndrome? (Capabilities, and opportunities) (Yes/No)
   1. ***If yes:***
      1. *Tell me more about it? Please describe it.*
      2. *What did you use it for?*
      3. *How did you use it?*
      4. *Are you still using it? Why not?*
      5. *How did you learn about it? What made you start using it?*
   2. ***If no:***
      1. *Have you ever heard of any digital tool that could help you in taking care of your metabolic syndrome? From whom?*

*OR: Are there any specific digital tools that you wish existed to help you in taking care of your metabolic syndrome?*

- - 1. *How do you think that tool could help you in taking care of your metabolic syndrome?*
    2. *Would you be interested in trying it? Why? Why not?*
    3. *Are there any specific reasons of why you have not used it yet? Please explain more.*

1. Can you tell me more about your experience in using [the digital tool that you mentioned]? *If answered no to the previous question: what do you know about the experience of the person who told you about the digital tool?* (Capabilities, opportunities, and motivations)
   1. *Did you/they find it helpful?*
      1. *Why? Why not?*
      2. *How?*
      3. *What did it help you/ them with in terms of managing metabolic syndrome?*
   2. *What were the things that were not helpful about it?*
      1. *How could it be improved?*
   3. *What did you/they like about it?*
   4. *What did you/they not like about it?*
   5. *Would you/they use it again? Why? Why not?*
   6. *Would you/they recommend it for others to use it? Why? Why not?*
   7. *How satisfied are/were you/they with it? Why?*
2. *In general, how do you feel about using digital tools to help in taking care of your health conditions?* (Capabilities, opportunities, and motivations)
   1. *Please explain more.*
   2. *How about people in your surroundings like family and friends?*
      1. *Do you know of any digital tools that they use?*
      2. *What do you know about their experience with it?*
      3. *Have they recommended it for you?*
         1. *What was your response? Why?*
      4. *Have you recommended any digital tools for them? Why? Why not?*
         1. *What was their response? Why?*
3. Can you tell me about any cultural, religious, or community beliefs that might influence how you use digital tools to manage your health? (Opportunities, and motivations)
   1. *Could you share an example of a belief or tradition that has affected your use of a digital tool?*
   2. *In what way did that belief or tradition influence your decision? Did it encourage or discourage you from using the tool?*
      1. *How would you change it to make it align with your belief or tradition?*
   3. *Have you ever chosen not to use a digital health tool because of these beliefs or traditions?*
4. What do you think would make it easier or more comfortable for people in your community to use digital tools to take care of their health? (Opportunities, and motivations)
   1. *Are there any specific features or functions that you think would make these tools more useful or appealing? Can you describe them?*
   2. *What kinds of support—like training, guidance, or community help—do you think people might need?*
   3. *How should information about a digital health tool be delivered to make it easy to understand and trustworthy?*

**The final set of questions focus on learning more about your background**: what is your:

- Age:
- Gender:
- Race/ ethnicity: *or country of origin*:
- Country of residence:
- Primary language:
- Occupation:
- In which of the following ranges does your annual household income fall?

For participants in the United States (USD) (0-25k, 25k-50k, 50k-75k, more than 75k); For participants in Saudi Arabia (SAR) (0-100k, 100k-200k, 200k-300,000, more than 300,000)

- **Closing Script**

Thank you so much for sharing your experiences with me today. That interview has concluded. Your insights are incredibly valuable and will help us better understand how people manage Metabolic Syndrome and use digital tools.
Before we finish, do you have any questions or anything else you’d like to add?
[Pause and allow time for any final remarks.]
Once again, thank you very much. This research would not be possible without your participation.

**[End of the Interview]**
